# Thymoquinone reduces cardiac damage caused by hypercholesterolemia in apolipoprotein E-deficient mice

**DOI:** 10.1186/s12944-018-0829-y

**Published:** 2018-07-26

**Authors:** Jingyi Xu, Liyue Zhu, Hongyang Liu, Mengye Li, Yingshu Liu, Fan Yang, Zuowei Pei

**Affiliations:** 1 Department of Endocrinology, Affiliated Zhongshan Hospital of Dalian University, No. 6 Jiefang Street, Dalian, China; 2 Rehabilitation Center, Zhejiang Hospital, No. 12 Lingyin Road, Hangzhou, Zhejiang China; 3 Department of Heart Intensive Care Unit, the First Affiliated Hospital of Dalian Medical University, No.193 Lianhe Road, Dalian, China; 4 Department of Special Medical Unit, the First Affiliated Hospital of Dalian Medical University, No. 193 Lianhe Road, Dalian, China; 5 Department of Endocrinology Dalian Municipal Central Hospital, No. 42 Xuegong Road, Dalian, China; 6 Department of Cardiology, Affiliated Zhongshan Hospital of Dalian University, No. 6 Jiefang Street, Dalian, 116001 China

## Abstract

**Background:**

Hypercholesterolemia is a well-established risk factor for cardiac damage, which can lead to cardiovascular diseases*.* Many studies have shown that thymoquinone protected rats from doxorubicin-induced cardiotoxicity and cardiac damage. The aim of this study was to investigate the possible protective effects of thymoquinone against cardiac damage in apolipoprotein E knockout (ApoE^−/−^) mice.

**Methods:**

Eight-week-old male ApoE^−/−^ mice were randomly divided into three groups: control group fed a normal diet (ND group), a high cholesterol diet (HD group) or HD mixed with thymoquinone (HD + TQ group). All groups were fed the different diets for 8 weeks. Blood samples were obtained from the inferior vena cava and collected in serum tubes. The samples were then stored at − 80 °C until used. Coronal sections of heart tissues were fixed in 10% formalin and then embedded in paraffin for histological evaluation. The remainder of the heart tissues was snap-frozen in liquid nitrogen for mRNA or immunohistochemical analysis.

**Results:**

The metabolic characteristics of total cholesterol (TC), low-density lipoprotein-cholesterol (LDL-c), and high-sensitivity C-reactive protein (hs-CRP) were lower in ApoE^−/−^HD + TQ mice than in ApoE^−/−^ HD mice. Lectin-like oxidized low-density lipoprotein receptor-1 (LOX-1) gene and protein expression was lower in the heart tissue of ApoE^−/−^HD + TQ mice than in those of ApoE^−/−^HD mice. Furthermore, the levels of macrophages and pro-inflammatory cytokines were lower in the cardiac tissues of ApoE^−/−^HD + TQ mice than in those of ApoE^−/−^HD mice.

**Conclusions:**

These results indicate that thymoquinone may provide a potential therapeutic target for cardiac damage caused by hypercholesterolemia.

## Background

ApoE^−/−^ mice are considered a well-accepted model of hypercholesterolemia [1]. Hypercholesterolemia leads to the development of cardiovascular diseases (CVD). These comprise disorders of the heart and blood vessels that cause various fatal events [2, 3].

In ApoE^−/−^ mice, hypercholesterolemia accelerates lipid deposition, atherosclerosis, and chronic inflammation [4, 5]. This process is enhanced in the presence of elevated levels of plasma lipids. Macrophages phagocytose oxidized lipids and form foam cells. Macrophage-derived foam cells release cytokines to recruit more macrophages to the lesions and influence lipid deposition [6]. However, the underlying pathophysiological mechanisms of the relationship between hypercholesterolemia and cardiac damage are not yet fully understood, especially in lipid deposition.

Recently, there has been a growing interest in using natural phytochemical compounds for alternative treatments of several conditions including cardiovascular diseases. Indeed, it has been estimated that at least 25% of the drugs used over the past few decades have been directly derived from plants and approximately 25% are chemically altered natural products [7]. Thymoquinone is one of these compounds. It is the main active ingredient of *Nigella sativa*, commonly known as black cumin or black seed, an annual flowering plant native to some areas such as the Mediterranean countries [8]. Since its first extraction in 1963 [9], thymoquinone has been shown to act as a potent free radical and superoxide scavenger [10–12]. In addition, it has been shown to have anti-inflammatory properties in in vivo and in vitro studies [13, 14]. It has also been shown to act as lipid peroxidation inhibitor and superoxide radical scavenger in doxorubicin (DOX)-induced cardiotoxicity in rats [15]. The aim of this study was to determine the role of thymoquinone in hypercholesterolemia-induced cardiac damage. Our results contribute to the understanding of the beneficial role and action mechanism of thymoquinone in hypercholesterolemia-induced cardiac disorders.

## Methods

### Animal studies

All animal studies were approved by the Animal Studies Committee of the affiliated Zhongshan Hospital of Dalian University. ApoE^−/−^ mice were purchased from Beijing Vital River Lab animal technology Co., LTD. (Beijing, China). All mice were maintained under constant conditions (temperature, 23–25 °C; humidity, 40–60%; 12 h light/dark cycle). At 8 weeks of age, the male mice were randomly divided into the following three groups: ApoE^−/−^ mice fed a normal diet which not contain cholesterol (*n* = 7), a high-cholesterol diet (*n* = 7) or thymoquinone (25 mg/kg/d; Sigma-Aldrich, St. Louis, MO, USA) in combination with a high-cholesterol diet (*n* = 7). The high-cholesterol diet contained 1.5% cholesterol and 15% fat. The experimental diet was purchased from the Shanghai Slac Laboratory Animal Co., Ltd. (Shanghai, China). After 8 weeks different diet, blood samples were obtained from the inferior vena cava and collected in serum tubes. The samples were then stored at − 80 °C until used. Coronal sections of heart tissues were fixed in 10% formalin and then embedded in paraffin for histological evaluation. The remainder of the heart tissues was snap-frozen in liquid nitrogen for mRNA or immunohistochemical analysis. All animal experiments were performed in accordance with the Guide for the Care and Use of Laboratory Animals. The study was approved by the ethical committee of the affiliated Zhongshan Hospital of Dalian University.

### Biochemical measurements

Serum was obtained and stored at − 80 °C. Total cholesterol, low-density lipoprotein cholesterol (LDL-c), and high-sensitivity C-reactive protein (hs-CRP) were measured using a Hitachi 7020 automatic analyzer (Hitachi, Tokyo, Japan).

### Haematoxylin and eosin staining (HE staining)

Cardiac tissues were fixed in 10% buffered formalin solution for 30 min and then dehydrated in 75% ethanol overnight, followed by paraffin embedding. Serial sections (4 μm) were subjected to haematoxylin and eosin (HE) staining for assessing the pathological changes.

### Periodic acid-Schiff staining

Cardiac tissues from each group were stored in 10% formalin, dehydrated in an ascending series of alcohol (75, 85, 90, and 100% alcohol, 5 min each) and then embedded in paraffin wax. The 4 μm-thick paraffin sections were sliced from these paraffin-embedded tissue blocks. Tissue sections were then de-paraffinized via immersion in xylene (3 times, 5 min each) and rehydrated using a descending series of alcohol (100, 90, 85, and 75% alcohol, 5 min each). Biopsy samples were stained using Periodic Acid-Schiff (PAS) stain to investigate changes in cardiac morphology and fibrosis. Red staining represented lipid depositon.

### Immunohistochemistry analysis

Immunohistochemistry was performed using the Histone Simple stain kits (Nichirei, Tokyo, Japan) according to the manufacturer’s instructions. Briefly, paraffin-embedded sections were deparaffinized with xylene and then rehydrated in a descending series of ethanol concentrations. The sections were treated for 15 min with 3% H_2_O_2_ in methanol to inactivate endogenous peroxidases and were then incubated at room temperature for 1 h with the primary antibodies against LOX-1 (rabbit anti-LOX-1 antibody, 1:200; Abcam, England) or CD68 (rabbit anti-CD68 antibody, 1:500; Abcam, England). All sections were analyzed using an Olympus B × 40 upright light microscope (Olympus, Tokyo, Japan). For each staining, totally 3 × 7 sections (7 mice) per group were analyzed and the representative images were presented. All image analyses were done by a blinded reviewer.

### RNA isolation and quantitative real-time RT-PCR

Total RNA was isolated from heart tissues using ISOGEN (Nippon gene, Tokyo, Japan) according to the manufacturer’s protocol. Complementary DNA (cDNA) was synthesized from total RNA using a first-strand cDNA synthesis kit (SuperScript VILO cDNA Synthesis Kit; Life Technologies, Carlsbad, CA, USA) according to the manufacturer’s instructions. Gene expression was quantitatively analyzed by real-time RT-PCR using fluorescent SYBR Green technology (Light Cycler; Roche Molecular Biochemicals). The cDNA amplification was performed as follows: the first cycle was maintained at 95 °C for 30 s, followed by 38 cycles consisting of denaturation (95 °C for 10 s), annealing (60 °C for 20 s), and extension (72 °C for 15 s). The LOX-1, SRA1,CD36, ABCA1,TNF-α and IL-6 were then processed using the 2 − ΔΔCt method, during which a single calibrated sample was compared against the gene expression of every unknown sample. The primer sequences are listed in Table 1.

**Table 1:** Primer oligonucleotide sequences

Gene	Primers
LOX-1	F:5′-CAAAGTCTCCCAACCAACCTGCAA-3′
	R:5′-ACATCCTGTCTTTCATGCGGCAAC-3′
SRA1	F:5′-GTTAAAGGTGATGGGGGACA-3
	R:5′-TCCCCTTCTCTCCCTTTTGT-3′
CD36	F:5′-CCTTAAAGGAATCCCCGTGT-3′
	R:5′-TGCATTTGCCAATGTCTAGC-3′
ABCA1	F:5′-AGCCAGAAGGGAGTGTCAGA-3′
	R:5′-CATGCCATCTGGGTAAACCT-3′
TNF-α	F:5′-TCTCATGCACCACCATCAAGGACT-3′
	R:5′-ACCACTCTCCCTTTGCAGAACTCA-3′
IL-6	F:5′-TACCAGTTGCCTTCTTGGGACTGA-3′
	R:5′-TAAGCCTCCGACTTGTGAAGTGGT-3′
β-actin	F:5′-CGATGCCCTGAGGGTCTTT-3′
	R:5′-TGGATGCCACAGGATTCCAT-3′

### Western blotting of heart tissues

Proteins were extracted from heart tissues using radioimmunoprecipitation assay buffer (P0013B; Beyotime, Shanghai, China). The samples were electrophoresed on 10% SDS-PAGE gels and proteins were transferred to polyvinylidene fluoride membranes (PVDF) (Immobilon, Millipore, Billerica, MA, USA). The membranes were blocked in Tris-buffered saline with 0.1% Tween-20 (TBS-T) containing 5% skim milk, then incubated in primary antibody diluents (P0023A; Beyotime), and gently shaken overnight at 4 °C. Primary antibodies against LOX-1 (rabbit anti-LOX-1 antibody, 1:250; Abcam), phospho-ERK (Rabbit anti-phospho-ERK, 1:1000; Cell Signalling Technology), and β-actin (1:1000; Cell Signalling Technology) were used. The membranes were then incubated with the secondary antibody (anti-rabbit Ig-G, 1:1000; Cell Signalling Technology) for 1 h. This analysis was independently performed three times. The protein levels are expressed as protein/β-actin ratios to minimize loading differences. The relative signal intensity was quantified using NIH Image J software.

### Statistical analysis

All data were presented as the mean ± SEM. Statistical analysis was performed using SPSS software version 23.0 (SPSS Inc., Chicago, IL, USA). Intergroup variation was measured using one-way ANOVA and subsequent Tukey’s test. The minimal level for statistical significance was set at *P* < 0.05.

## Results

### Metabolic characterization

**Fig. 1:**
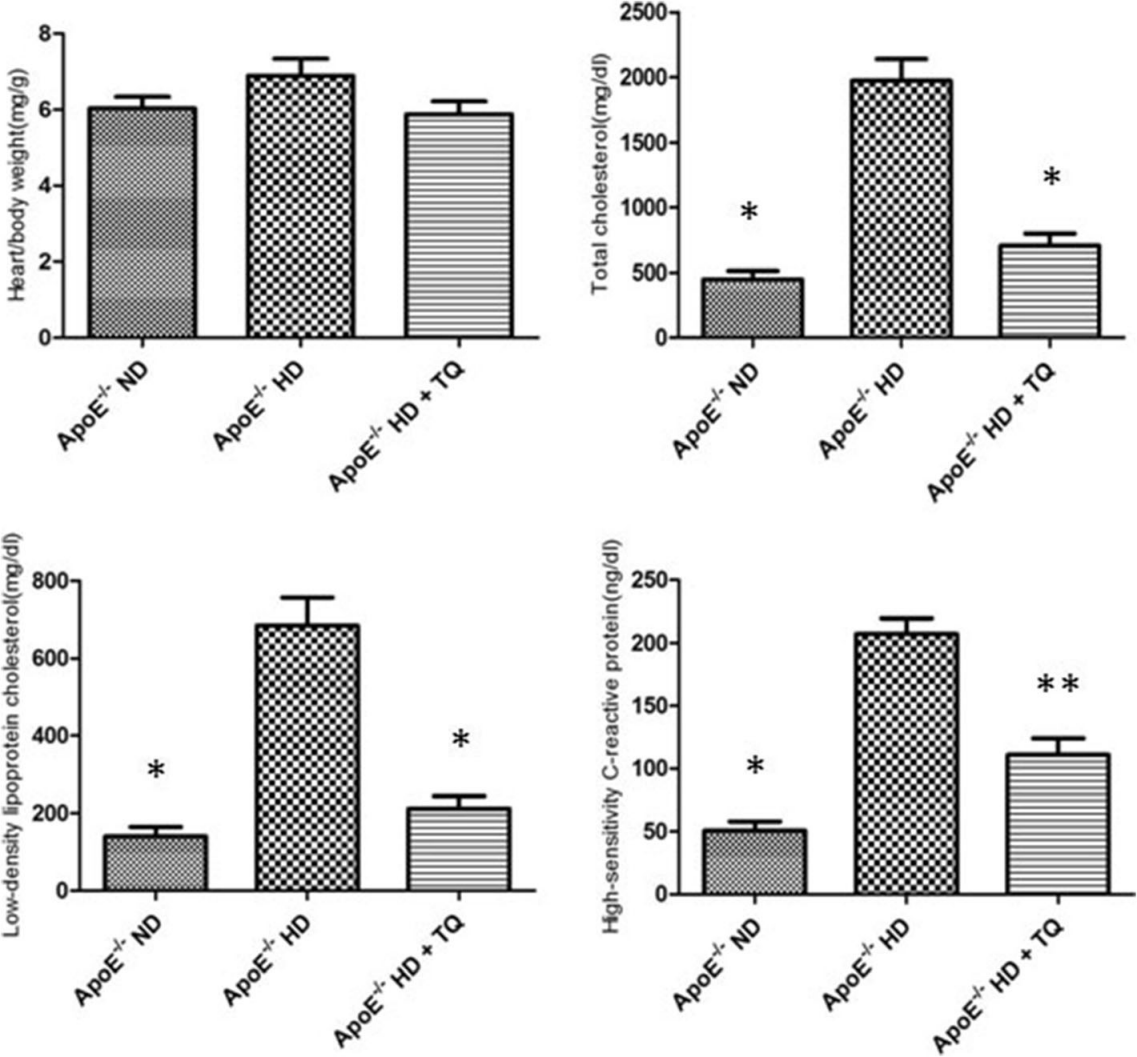
Metabolic data from the three mice groups after 8 weeks of different diets. Heart/body weights*,* total cholesterol, low-density lipoprotein, and high-sensitivity C-reactive protein of three groups after 8 weeks of different treatments are presented here. Data are means±SEM; *n* = 6–7 per group. *P < 0.01 vs ApoE^−/−^HD; **P < 0.05 vs ApoE^−/−^HD

The metabolic characteristics of ApoE^−/−^ mice after 8 weeks of dietary treatment are summarized in Fig. 1. Heart/body weights did not differ among the three groups. The ApoE^−/−^HD mice group showed markedly increased total cholesterol and low-density lipoprotein levels, but these significantly decreased in the HD + TQ group*.* There was no difference between the HD + TQ group and the control group. Hs-CRP levels significantly decreased in the HD + TQ group compared to that in the HD group.

### Thymoquinone reduced lipid deposition on histological assessments in heart structure of ApoE^−/−^ mice fed HD

**Fig. 2:**
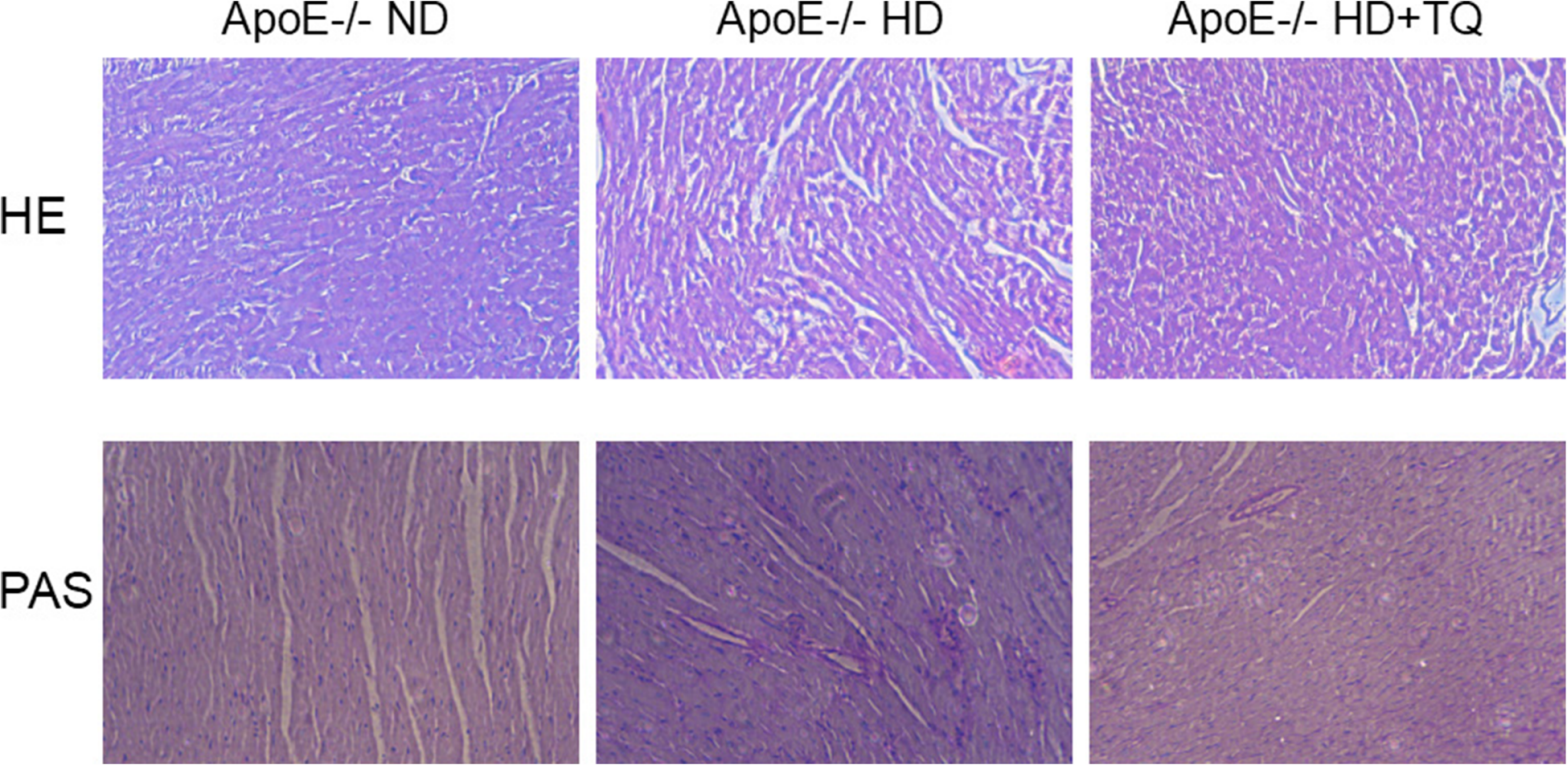
HE and PAS staining in heart tissues of three groups. Thymoquinone could ameliorate inflammatory cells infiltration and lipid deposition in ApoE^−/−^ HD + TQ group mice. Magnification 40 X

HE and PAS staining facilitated the visualization of cardiac tissular disorder, inflammatory cells infiltration and massive fibrosis with cardiac damage of hypercholesterolemia. Treatment with thymoquinone was helpful for ameliorating inflammatory cells infiltration and lipid deposition in ApoE^−/−^ HD + TQ group mice compared to ApoE^−/−^ HD group mice (Fig. 2).

### Thymoquinone reduced LOX-1 gene expression in the heart tissue of ApoE^−/−^ mice fed HD

**Fig. 3:**
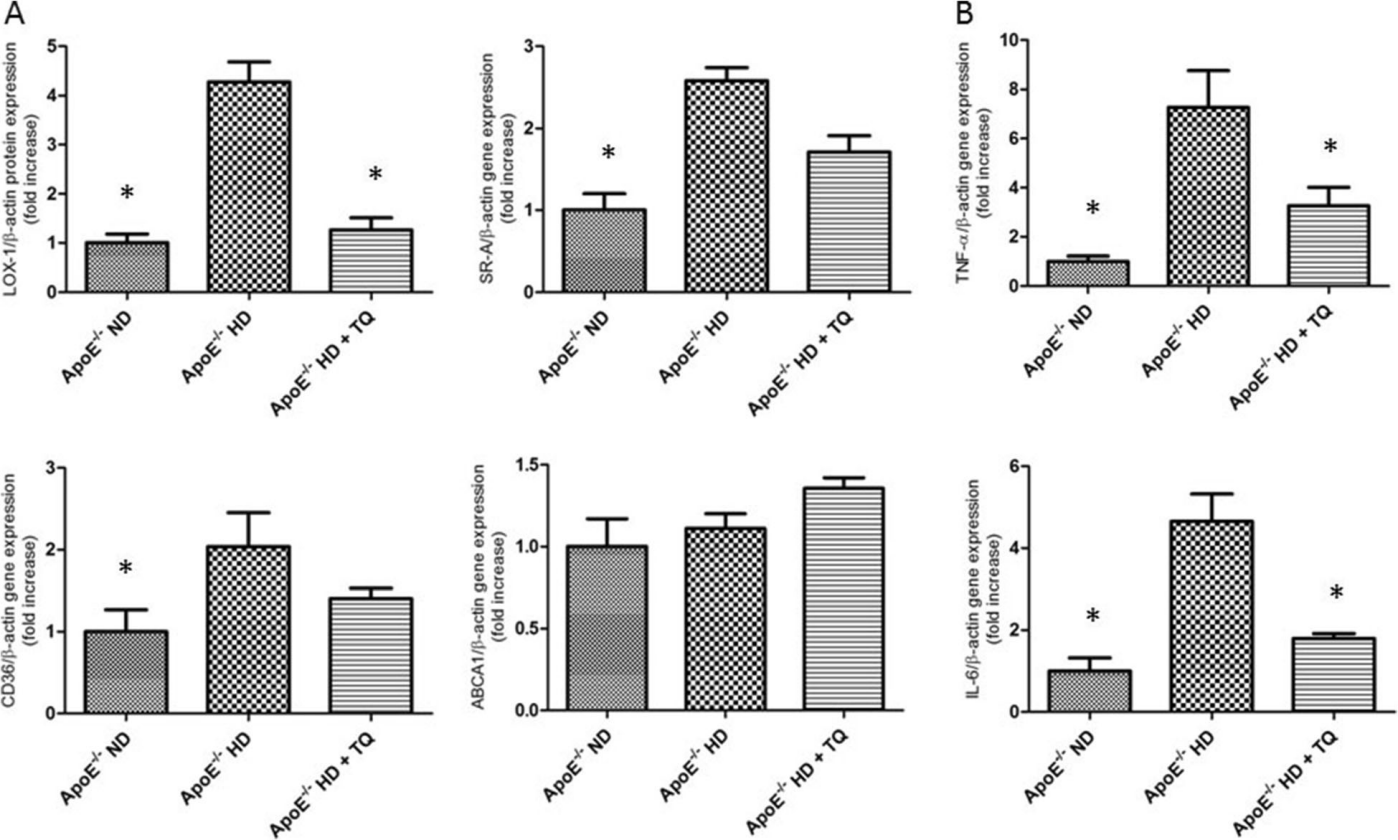
Scavenger receptors and pro-inflammatory gene expression in the heart tissue of the three mice groups after 8 weeks with different diets. **a** Relative mRNA expression of LOX-1, SRA, CD36, and ABCA1 in the heart tissue of each group after 8 weeks of feeding the mice with different diets. **b** Relative mRNA expression of TNF-α and IL-6 in the hearts tissue of each groups after 8 weeks with different treatments. Data are given as the means ± SEM; *n* = 6 in each group. * *P* < 0.01

To investigate the mechanism of lipid accumulation in the heart, gene expression of relevant receptors and the TP-binding cassette transporter A1 (ABCA1) was examined by RT-PCR. LOX-1 gene expression significantly increased in the heart tissue of ApoE^−/−^ HD group mice compared to that in control mice. The increased expression of LOX-1 was suppressed in the ApoE^−/−^ HD + TQ group. Expression of scavenger receptor-class A (SR-A) and CD36 increased in ApoE^−/−^HD mice compared to that in the control group. However, the levels were similar to those of ApoE^−/−^ HD + TQ mice. Expression of ABCA1 did not differ among the three groups (Fig. 3a). These results suggest that LOX-1, SR-A, and CD36 influence lipid accumulation in the heart tissue of ApoE^−/−^HD mice. LOX-1, in particular, appears to be a critical factor for mitigation of lipid accumulation in the heart tissue of ApoE^−/−^HD + TQ mice compared to that in ApoE^−/−^HD mice.

### Thymoquinone reduced tumor necrosis factor (TNF)-α and interleukin (IL)-6 gene expression in the heart tissue of ApoE^−/−^ mice fed HD

To examine the involvement of pro-inflammatory cytokines in hypercholesteremic cardiac damage, IL-6 and TNF-α gene expression was measured by real-time PCR. Both IL-6 and TNF-α were up-regulated in ApoE^−/-^HD mice. However, this up-regulation was attenuated in ApoE^−/-^HD + TQ mice (Fig. 3b).

### Thymoquinone reduced LOX-1 expression in the hearts tissue with immunohistochemistry

**Fig. 4:**
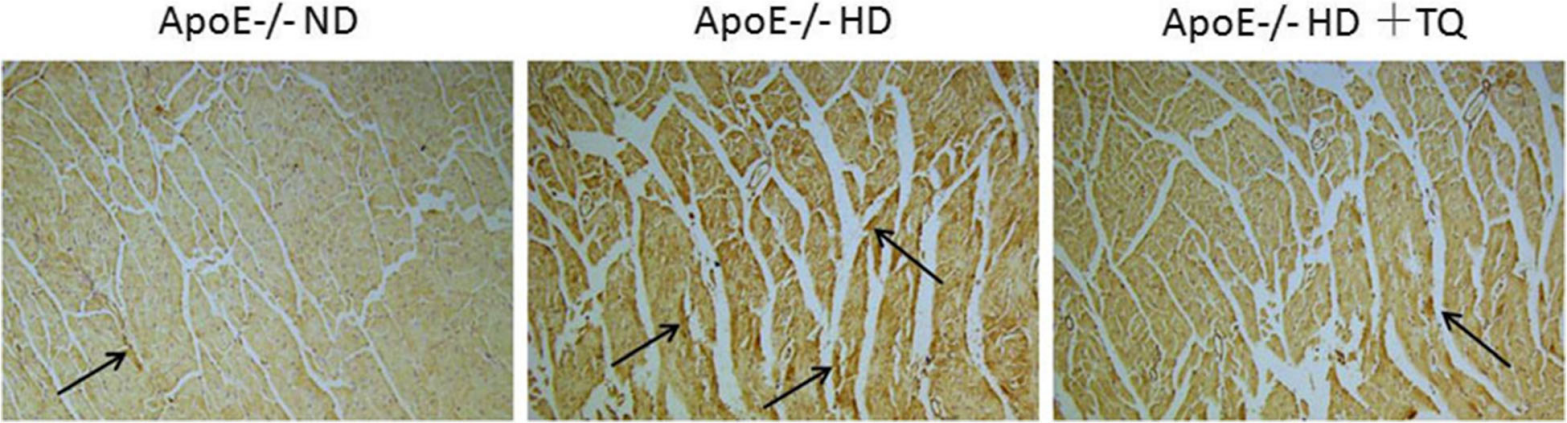
LOX-1 expression in the heart tissue of the three groups after 8 weeks with different treatments. Representative immunohistochemistry staining for LOX-1 in heart tissue of mice fed with different diets. Magnification 40 X. Arrows indicate positively stained cells

To evaluate LOX-1 expression in the heart tissue, LOX-1 immunostaining was performed (Fig. 4). The HD + TQ group mice showed a markedly reduced LOX-1 protein expression in the heart tissue compared to ApoE^−/−^HD mice. These results indicate that thymoquinone reduced LOX-1 protein expression in ApoE^−/−^HD mice.

### Thymoquinone reduced macrophages in the heart tissue of ApoE^−/−^ mice in the HD group

**Fig. 5:**
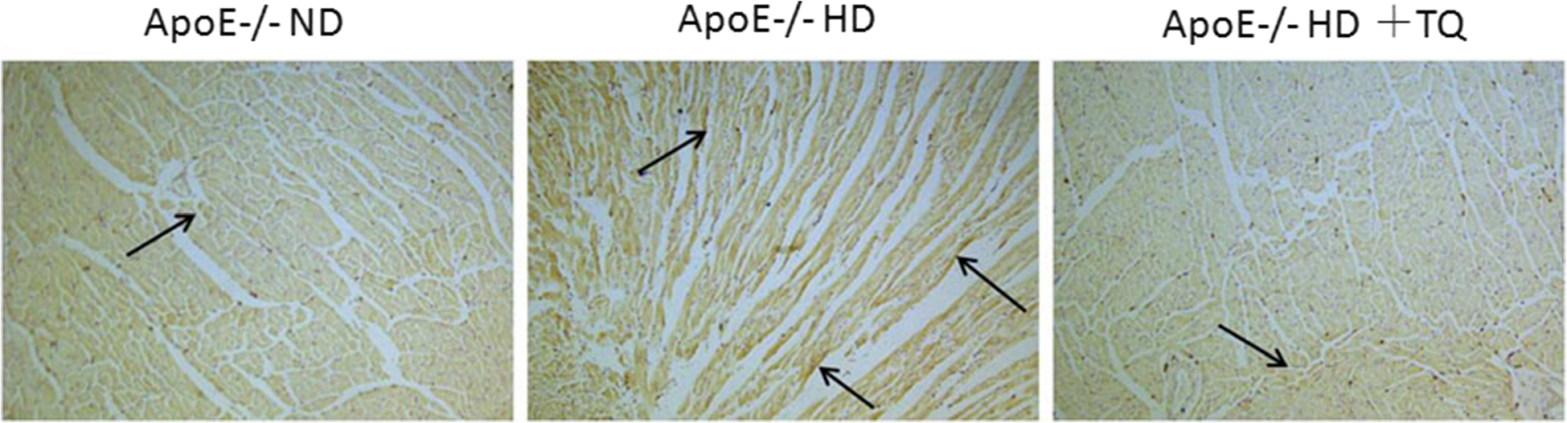
CD68 expression in the hearts of the three groups after 8 weeks with different treatments. Representative immunohistochemistry staining for CD68 expression in heart. Magnification 40 X. Arrows indicate positively stained cells

To detect infiltrating macrophages, immunohistochemical analysis using CD68 was performed (Fig. 5). The mice in the HD + TQ group showed a markedly reduced CD68-positive staining in the heart compared to ApoE^−/−^HD mice. These results indicate that thymoquinone reduced macrophage infiltration in the ApoE^−/−^ HD mouse hearts.

### Thymoquinone reduced LOX-1 protein expression in the hearts of ApoE^−/−^HD mice

**Fig. 6:**
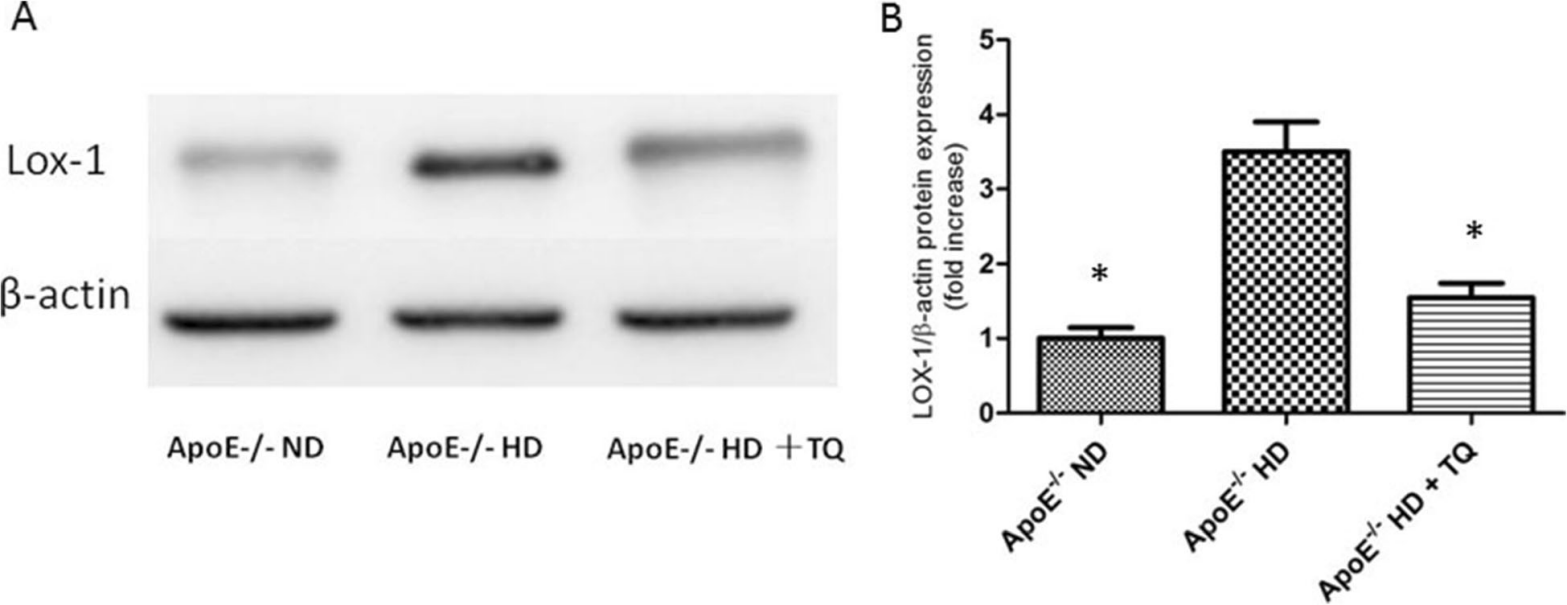
LOX-1 protein expression in the heart tissue of the three groups after 8 weeks with different treatments. **a** Immunoblotting for LOX-1 protein expression in hearts tissue. **b** The bar graph shows the quantification of LOX-1 protein expression. Data are given as the means±SEM; *n* = 3 in each group. * *P* < 0.05 vs ApoE^−/−^HD

To evaluate LOX-1 protein expression in the heart tissue, LOX-1 protein immunoblotting was performed (Fig. 6a). We found that the LOX-1 protein levels in ApoE^−/−^HD + TQ mice were significantly suppressed compared to that in the ApoE^−/−^HD group (Fig. 6b)*.*

### Thymoquinone reduced phospho-ERK levels in the hearts tissue of ApoE^−/−^HD mice

**Fig. 7:**
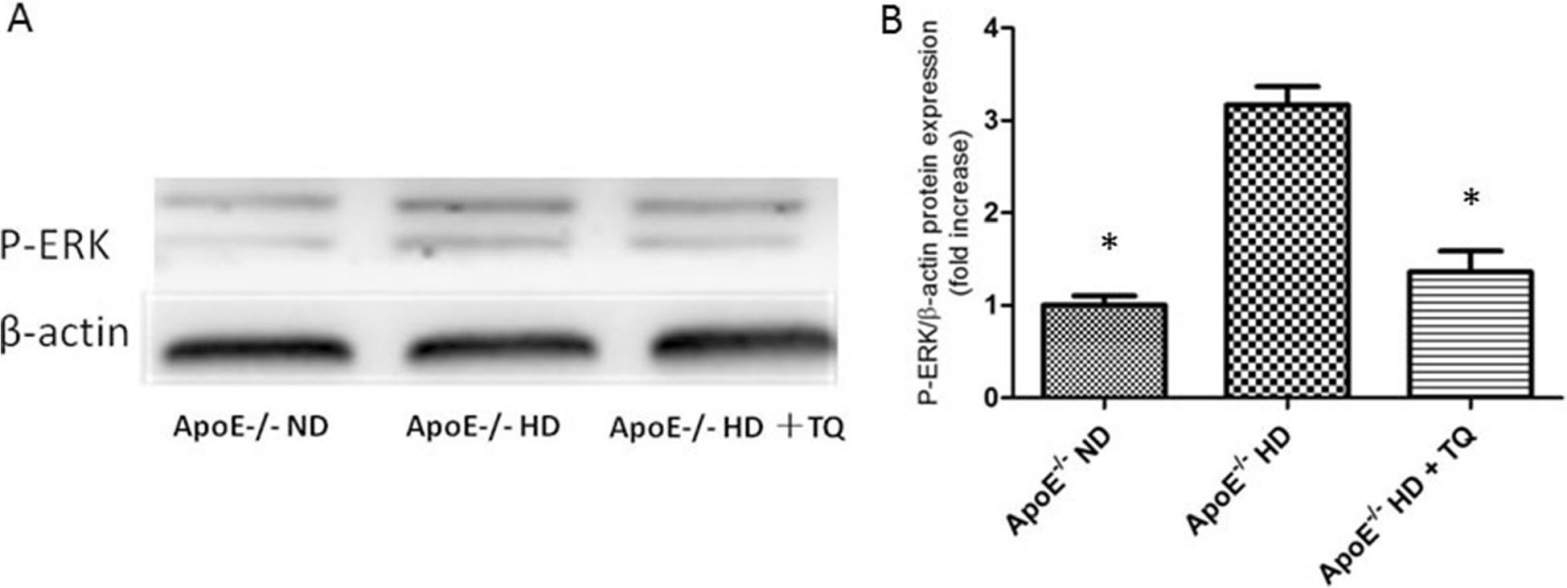
Phosphor-ERK levels in the hearts of the three groups after 8 weeks with different treatments. **a** Immunoblotting for phosphor-ERK levels in heart tissues. **b** The bar graph shows the quantification of phosphor-ERK levels. Data are given as the means±SEM; *n* = 3 in each group. *P < 0.05 vs ApoE^−/−^HD

Protein kinases play a role in foam cell formation, lipid deposition, and phosphorylation of -ERK. To analyze the phosphorylation of ERK, protein immunoblotting was performed (Fig. 7a). We found that the phosphorylation level of ERK in ApoE^−/−^HD + TQ mice was significantly suppressed compared to that in ApoE^−/−^HD mice (Fig. 7b)*.*

## Discussion

This study demonstrates that thymoquinone has a protective effect on cardiac damage, which against progressive of lipid deposition, pro-inflammatory cytokine, and macrophage accumulation secretion elicited by hypercholesterolemia.

According to the metabolic characteristics, we found that TC and LDL-c increased in the HD mouse group compared with the ND group of ApoE^−/−^ mice. These results are in agreement with the reports by Daniel Kolbus [16]. Interestingly, TC and LDL-c were significantly suppressed in the HD + TQ group compared to that in the HD group. Several clinical studies have indicated that the hs-CRP level reflects the instability of atherosclerotic lesions and it can be used as a biomarker for risk prediction of cardiovascular events [17–20]. Our results indicate that thymoquinone influences the cholesterol metabolism and hs-CRP. However, further studies are needed to clarify the mechanisms.

Hypercholesterolemia is a major independent risk factor of cardiac damage. Hyperlipidemia promotes lipid deposition and inflammation in the aorta [21, 22]*.* In our study, we observed cardiac tissular disorder, inflammatory cells infiltration and massive fibrosis with cardiac damage of hypercholesterolemia by HE and PAS staining. There were less inflammatory cells infiltration and lipid deposition in ApoE^−/−^ HD + TQ group mice compared to ApoE^−/−^ HD group mice. Cellular lipid homeostasis involves regulation of the influx, synthesis, catabolism, and efflux of lipids. An imbalance of these processes can result in the conversion of macrophages and vascular smooth muscle cells into foam cells. This process is mediated by several independent pathways, including SR-A, class B (CD36), and LOX-1 [23–25]*.* Pro-inflammatory genes (TNF-α and IL-6) were reported to be expressed at high levels and to contribute to cardiovascular diseases caused by hyperlipidemia [26, 27]*.*

### Mechanism of action of thymoquinone

Thymoquinone is a potent phytochemical anti-oxidant due to its scavenging activity against several ROS including superoxide anions, hydroxyl radicals, and singlet molecular oxygen, thus it can antagonize the adverse effects resulting from elevated ROS levels in various disorders [15, 28]. It has been shown that thymoquinone and tert-butylhydroquinone (TBHQ), a structurally related synthetic compound, can robustly inhibit iron-dependent microsomal lipid peroxidation. The anti-oxidative potential of thymoquinone may be related to the redox properties of its quinone moiety and its unrestricted ability to cross physiological barriers and access subcellular compartments, all of which help its radical scavenging effects [29, 30].

These effects include the reduction of total blood cholesterol and lipid peroxidation levels during cardiac damage [21, 22, 27]*.* In a previous study, thymoquinone acted as an inhibitor of lipid peroxidation and superoxide radical scavenger in DOX-induced cardiotoxicity in rats. Moreover, thymoquinone protected against cypermethrin-induced necrosis, degeneration, and loss of striation in the heart. It resulted in a reversal of cypermethrin-induced oxidative stress and lipid peroxidation [15, 31].

### Thymoquinone affects gene expression

In our study, we analyzed the gene expression of scavenger receptors including SR-A, CD36, and LOX-1. We found that LOX-1 gene expression was suppressed in the HD + TQ group of mice*.* LOX-1 was originally identified in endothelial cells. It is a 50-kDa type II membrane glycoprotein that contains a short N-terminal cytoplasmic domain, a single transmembrane domain, a short neck or stalk region, and an ox-LDL-binding C-terminal extracellular C-type lectin-like domain. On the cell surface, LOX-1 consists of 3 homodimers that are bound to ox-LDL, and it plays an important role in ox-LDL uptake and foam cell formation [32, 33]. In contrast, deletion of LOX-1 has been shown to reduce the uptake of oxidized LDL and inhibit atherosclerosis in mice fed a high-cholesterol diet [34]. Therefore, suppression of LOX-1 expression in ApoE^−/−^HD + TQ mice may reduce foam cell formation. Thymoquinone also reduced LOX-1 protein expression in kidney tissues of ApoE^−/−^HD mice. Protein kinases regulate foam cell formation and lipid deposition. As shown earlier, enhanced LOX-1 expression was attenuated by inhibitors of ERK, PKC, and NF-κB, indicating that the increased production of intracellular ROS and activation of the PKC/MAPK pathway are the initial signaling events in LOX-1 gene regulation [35]. Our results show that phosphorylation of ERK was significantly reduced in the HD + TQ group compared to that in the HD group. We speculated that thymoquinone regulates LOX-1 via the phospho-ERK pathway.

### The role of thymoquinone in inflammation

Pro-inflammatory genes (TNF-α and IL-6) have been reported to be expressed at high levels and contribute to kidney injury in hyperlipidemia [27, 36]*.* Furthermore, TNF-α and IL-6 were shown to induce LOX-1 up-regulation in smooth muscle cells [37]*.* The present study shows that TNF-α and IL-6 gene expressions were reduced in the HD + TQ group compared to the HD group. Attenuation of TNF-α and IL-6 expression may have also reduced LOX-1 expression in ApoE^−/−^HD + TQ mice. It has been reported that thymoquinone reduces TNF-*α* and IL-6 in blood and arthritis tissues. In addition, it protects tissues by reducing inflammation [13, 14].

Cardiac damage induced by hyperlipidemia is usually associated with an increase in the number of macrophages. Macrophage-derived foam cells release cytokines that recruit more macrophages to lesions and influence lipid deposition [6]. The marker CD68 identifies macrophages. CD68-positive cells were found in liver tissue damaged by hyperlipidemia [38]. In the present study, immunohistochemical staining with anti-CD68 antibody showed that CD68-positive cells significantly increased in the HD group compared to that in the ND group of ApoE^−/−^ mice. However, mice in the HD + TQ group showed markedly reduced accumulation of CD68-positive cells in the heart tissue compared to mice of the HD group. This indicates that thymoquinone reduces macrophage accumulation in mice of the HD group.

## Conclusions

Our study establish that thymoquinone contributes to the mitigation of hypercholesterolemic cardiac damage as shown by the downregulation of LOX-1 and the suppression of foam cell formation, lipid deposition, and macrophage accumulation. These findings provide new insights for the role of thymoquinone in hypercholesterolemia-induced cardiac damage and raise the possibility of a novel therapeutic intervention for the treatment of the progression of cardiovascular diseases.

## Abbreviations

ABCA1: ATP-binding cassette transporter A1
Hs-CRP: high-sensitivity C-reactive protein
IL-6: interleukin- 6
LDL-c: low-density lipoprotein cholesterol
LOX-1: lectin-like oxidized low-density lipoprotein receptor-1
SRA: scavenger receptor-A
TC: total cholesterol
TNF-α: tumor necrosis factor-α
